# Clinicopathological analysis of 134 patients with squamous cell carcinoma of the mandibular gingiva

**DOI:** 10.1016/j.heliyon.2023.e23120

**Published:** 2023-11-30

**Authors:** Hirofumi Tomioka, Naoto Nishii, Yu Oikawa, Takuma Kugimoto, Takeshi Kuroshima, Hideaki Hirai, Kou Kayamori, Atsushi Kaida, Masahiko Miura, Hiroyuki Harada

**Affiliations:** a Department of Oral and Maxillofacial Surgical Oncology, Division of Oral Health Sciences, Graduate School of Medical and Dental Sciences, Tokyo Medical and Dental University, Japan; b Department of Oral Pathology, Division of Oral Health Sciences, Graduate School of Medical and Dental Sciences, Tokyo Medical and Dental University, Japan; c Department of Dental Radiology and Radiation Oncology, Division of Oral Health Sciences, Graduate School of Medical and Dental Sciences, Tokyo Medical and Dental University, Japan

**Keywords:** Squamous cell carcinoma, Oral cancer, Mandibular gingiva, Risk factor

## Abstract

**Objective:**

The accurate assessment of the involvement of mandibular gingival squamous cell carcinoma (SCC) is essential for determining the extent of resection and is also useful for predicting lymph node metastasis and prognosis. The purpose of this study was to investigate the factors for predicting the prognosis.

**Study design:**

We reviewed 134 patients with mandibular gingival SCC treated between 2008 and 2017. The clinical findings, TN stage, relationship between radiographical type and histological pattern, and factors affecting the survival rate were investigated.

**Results:**

The moth-eaten radiographic type was significantly associated with histologically infiltrative pattern. For all 134 cases, the 5-year OS was 89.5 %, and 5-year DSS was 93.9 %. The 5-year DSS was 95.0 % for cN0 and/or pN0 cases and 90.3 % for pN (+) cases, with a significant difference. The significant risk factors for lymph node metastasis were teeth extractions by previous physicians and moth-eaten radiographic type.

**Conclusion:**

The risk factor for poor prognosis was lymph node metastasis. In addition, teeth extractions by previous physicians and moth-eaten radiographic type were the risk factors for lymph node metastasis. It is recommended that these cases be treated considering the possibility of cervical lymph node metastasis.

## 1 Introduction

Squamous cell carcinoma (SCC) of the mandibular gingiva shows a tendency to invade the mandible bone because of its anatomical features. Therefore, surgical resection including mandible bone is generally required. The goal of surgical treatment is complete resection of the tumor. In addition, preservation of oral function is an important determinant of surgery nowadays [1]. The location of tumor invasion should be accurately estimated preoperatively not only for determining the necessary extent of resection but also for predicting cervical lymph node metastasis and the prognosis. Several factors affecting the prognosis of mandibular gingival SCC have reported; as follows, extractions of teeth in the tumor site performed before initial treatment, radiographical type of mandibular resorption, and histological pattern of bone invasion [[2], [3], [4]]. Histological cervical lymph nodes metastasis has also been reported as a poor prognostic factor [5]. The purpose of this study was to statistically investigate the factors for predicting the prognosis among clinical, imaging, and histopathological features in mandibular gingival SCC. Accordingly, by a comprehensive examination of these factors, we analyzed the relationships among the radiographical type of mandibular resorption, histological pattern of bone invasion, and cervical lymph node metastasis in mandibular gingival SCC and its relation with the prognosis. We believe that these findings will be valuable in determining the characteristics of mandibular gingival SCC. The results of this study will contribute to the appropriate treatment of mandibular gingival SCC.

## 2 Materials and methods

### 2.1 Study design and patient selection

This retrospective study included 145 patients with mandibular gingival SCC treated at the Department of Oral and Maxillofacial Surgical Oncology in Tokyo Medical and Dental University between 2008 and 2017. After excluding cases with secondary tumors, distant metastasis at initial treatment, metastasis to the oral cavity, palliative treatment, and lack of adequate follow-up data, the final study sample consisted of 134 patients.

### 2.2 Clinical data collection

**Fig. 1:**
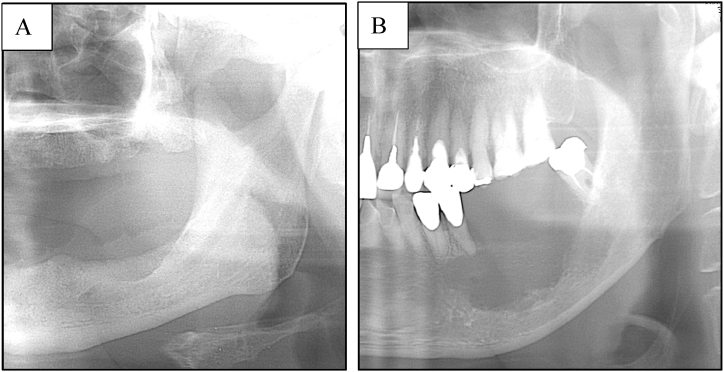
The radiographical type of mandibular resorption. A: pressure-type, B: moth-eaten type.

**Fig. 2:**
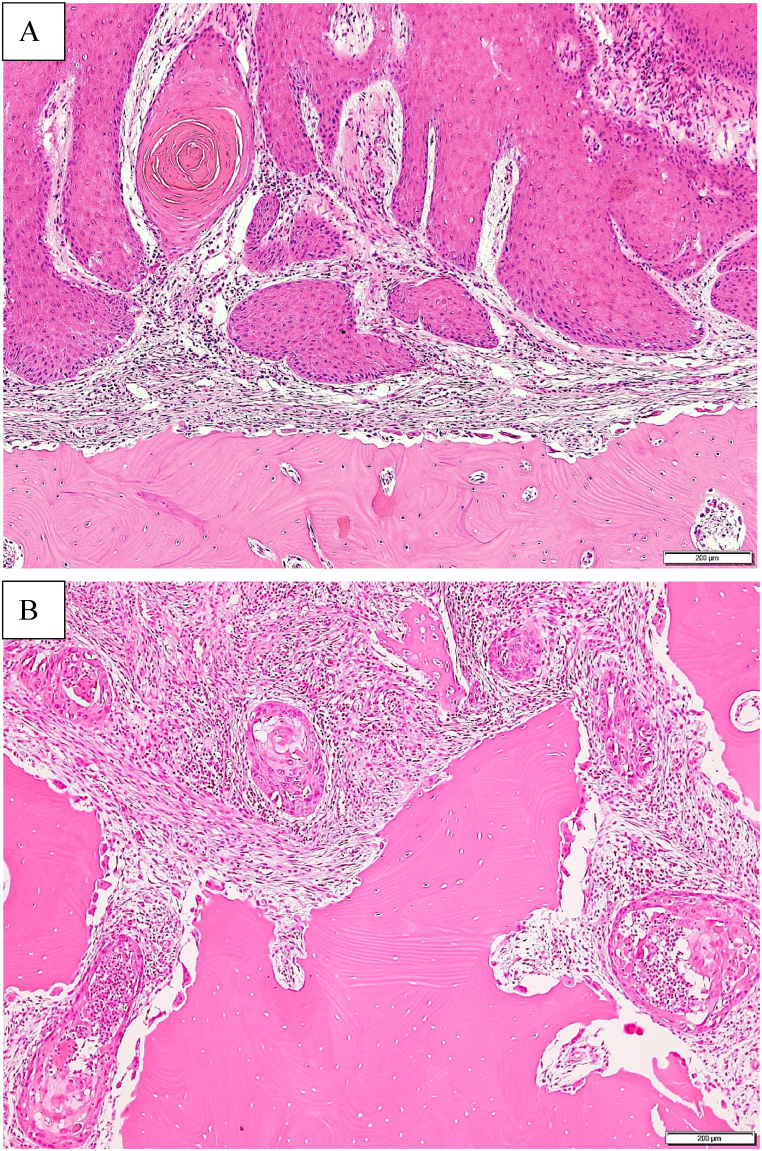
The histological pattern of bone invasion. A: erosive pattern, B: infiltrative pattern.

We investigated the clinical findings, TN stage, methods of primary tumor excision, relationship between radiographical type of mandibular resorption and histological pattern of bone invasion, histological differentiation and factors affecting the survival rate. T stages T1, T2, and T3 were determined according to the UICC/AJCC staging system (7th edition) based on size. The classification of mandibular canal invasion proposed by Ebrahimi et al. [6] and Izumo et al. [7] was further used to define T4 stage as the lesions transcending the mandibular canal in the molar region or the imaginary line connecting the bilateral mental foramens in the anterior region [8]. The radiographical type of mandibular resorption was evaluated on panoramic radiograph. According to the classification suggested by Swearingen et al. [9], the patients were classified into the following three groups: no bone resorption, pressure-type, and moth-eaten type (Fig. 1 A, B). The histological pattern of bone invasion was classified into the following three groups according to the classification proposed by Slootweg et al. [10]: no bone invasion, erosive pattern, and infiltrative pattern (Fig. 2 A, B). The radiological and pathological evaluations were performed with more than 10 years of experience.

### 2.3 Statistical analysis

The chi-squared tests were used to analyze any relevant differences. Survival rates were calculated using the Kaplan-Meier method, and the log-rank test was performed. Multivariate analysis using logistic regression was subsequently performed to evaluate variables that were significantly different in the univariate analysis. The *p* values less than 0.05 were considered statistically significant. All statistical analysis were performed using SPSS Statistics version 25 for Windows (SPSS Japan Inc., Tokyo, Japan).

The Institutional Review Board of the Faculty of Hospital of Tokyo Medical and Dental University approved this clinicopathological study and waived the requirement for written informed patient consent owing to the retrospective study design (approval No. D2015-600). The authors confirm that all experiments were conducted in accordance with the relevant guidelines and regulations.

## 3 Results

### 3.1 Patient characteristics and treatments

The clinical characteristics of the patients are shown in Table 1. Among the 134 patients, 75 were male and 59 were female. The average age of the patients was 68.5 years (range, 43–93 years). The TN stage was determined at the first visit; T4 was the most common and was noted in 53 patients, followed by T2 in 51, and N0 was in 106 patients. Surgery was the initial treatment method for all patients. The duration of follow-up ranged from 1.5 months to 162 months, with an average of 67 months.

**Table 1:** Clinicopathological characteristics of patients.

Characteristics	Patients no.
Sex	
Male	75
Female	59
Mean age (range)-yr	68.5 (43–93)
T stage	
T1	21
T2	51
T3	9
T4a	51
T4b	2
N stage	
N0	106
N1	12
N2b	14
N2c	2
Histological differentiation	
Well	81
Moderately	48
Poorly	5
Postoperative therapy	
No	115
Radiotherapy	14
Chemotherapy	1
Chemoradiotherapy	4

The methods of primary tumor excision according to the T stage are shown in Table 2. Most patients with T1 tumors underwent gingivectomy or marginal mandibulectomy, and the extent of resection increased as the T stage progressed. Twenty-one patients with T1 or T2 tumors underwent segmental mandibulectomy or hemimandibulectomy. The reason for this was the following cases: patients whose teeth included in the tumors were extracted by previous physicians, patients with the moth-eaten radiographical type of mandibular resorption, and older edentulous patients with thin mandibles and limited amount of bone left after marginal mandibulectomy.

**Table 2:** Excision of primary tumors according to T stage.

	Gingivectomy	Mandibulectomy				Total
		Marginal	Segmental	Hemi	Subtotal	
T1	6	14	1			21
T2	1	30	19	1		51
T3		4	5			9
T4a			40	10	1	51
T4b				2		2
Total	7	48	65	13	1	134

Among the 134 patients, 109 patients underwent neck dissection as the initial treatment. Therapeutic neck dissection was performed in 28 patients of these patients and elective neck dissection was performed in 81 patients. Of the 81 patients who underwent elective neck dissection, 12 patients underwent neck dissection for primary tumor resection by cervical approach and 69 patients underwent neck dissection for reconstruction with free tissue transfer. Among the 109 patients who underwent neck dissection, 32 patients had histological cervical lymph node metastasis and 77 patients had no evidence of lymph node metastasis. Among the 25 patients who did not undergo neck dissection, two patients developed subsequently cervical lymph node metastasis and 23 did not show any evidence of lymph node metastasis.

Additionally, 19 patients with close or positive margin of the primary tumor on the histological specimen or histological lymph node metastases in four or more cervical lymph nodes were treated by the 50–66 Gy postoperative irradiation and/or chemotherapy with platinum-based anticancer agents.

### 3.2 Radiographical type and histological pattern

The radiographical type of mandibular resorption was determined on panoramic radiograph. No bone resorption or pressure-type bone resorption was observed in 75 patients (56.0 %) and moth-eaten type of bone resorption in 59 cases (44.0 %). Histologically, 73 patients (54.5 %) showed no bone invasion or erosive pattern of bone invasion and 61 patients (45.5 %) showed infiltrative pattern of bone invasion. Statistically, the moth-eaten type of bone resorption on radiographic examination was significantly associated with the infiltrative pattern of bone invasion on histological examination (*p* < .001) (Table 3).

**Table 3:** Radiographical type of mandibular resorption and histological pattern of invasion.

	Radiographical type		Total
	No or Pressure	Moth-eaten	
Histological pattern No or Erosive	60	13	73
Infiltrative	15	46	61
Total	75	59	134

Significant at *p* < .001.

### 3.3 Clinical results

**Fig. 3:**
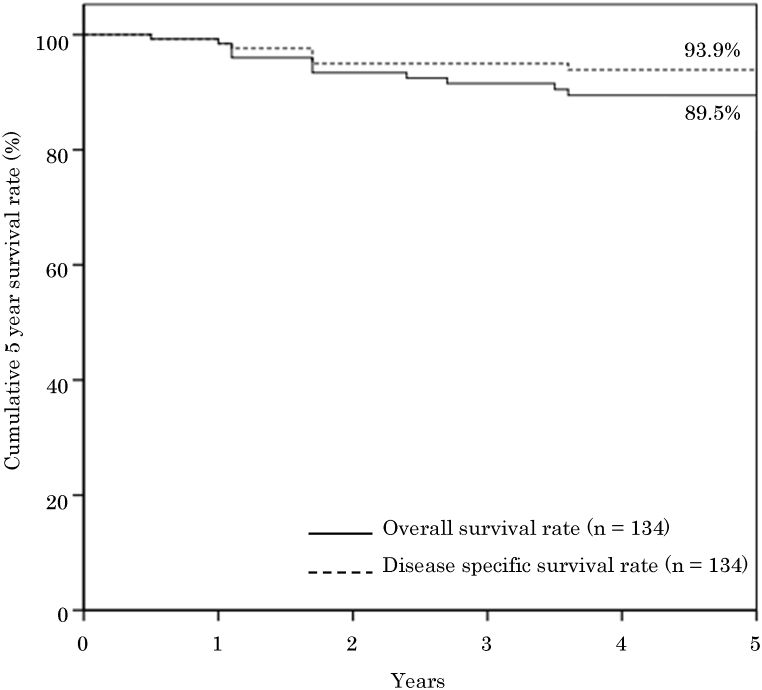
Cumulative 5-year survival rates.

The treatment outcomes in patients included disease-free survival in 117 patients, death caused by cancer in nine (primary tumor-related death in two, cervical metastasis-related death in one, and distant metastases-related death in six), and death caused by other causes in eight. The 5-year cumulative survival rate for all 134 patients included 89.5 % overall 5-year survival rate and 93.9 % disease-specific 5-year survival rate, which appeared to be good outcomes (Fig. 3). The disease-specific 5-year survival rate according to cervical lymph node metastasis status was 95.0 % for cN0 and/or pN0 cases (n = 100), while 90.3 % for pN (+) cases (n = 34); a significant difference was observed between the two groups (*p* = .036). The disease-specific 5-year survival rates and results of log-rank tests for other factors are presented in Table 4. No significant differences were observed except for the presence of cervical lymph node metastases.

**Table 4:** Disease specific 5-year survival rate (Log-rank test).

	Variables	Survival rate (%)	*p* value
Sex	Male	90.5	.194
	Female	98.1	
Age	≦ 68	92.7	.649
	≧ 69	95.4	
Teeth extraction by previous physician	None	95.6	.085
	Done	90.7	
T stage	T1・2	98.5	.237
	T3・4	88.8	
Radiographical type	No or Pressure	96.8	.203
	Moth-eaten	90.4	
Histological pattern	No or Erosive	96.6	.289
	Infiltrative	90.9	
Histological differentiation	Well	92.8	.873
	Moderately/Poorly	95.7	
Lymph node metastasis	cN0 and/or pN0	95.0	.030^a^
	pN(+)	90.3	

a Significant at *p* < .05.

### 3.4 Risk factors for cervical lymph node metastasis

To found that the prognosis of mandibular gingival SCC is determined by the presence of cervical lymph node metastasis, we analyzed the risk factors for cervical lymph node metastasis. Statistical analysis was performed for sex, age, teeth extraction by previous physicians, T stage, radiographic type of mandibular resorption, and histological pattern of bone invasion and histological differentiation. In univariate analysis, patients whose teeth in the tumors were extracted by previous physicians and moth-eaten type of radiographic mandibular resorption showed significantly higher rates of cervical lymph node metastasis (Table 5). Multivariate analysis using logistic regression was subsequently performed to evaluate variables affecting lymph node metastasis that were significantly different in the univariate analysis. The results of the multivariate analysis also showed that extractions of the teeth in the tumors by previous physicians (odds ratio (OR), 2.078; 95 % confidence interval (CI), 1.015–4.973), and moth-eaten type of radiographic mandibular resorption (OR, 4.298; 95 % CI, 1.733–10.656) were risk factors for cervical lymph node metastasis (Table 6).

**Table 5:** Results of the univariate analysis in risk factors for cervical lymph node metastasis.

	Variables	Lymph node metastasis		*p* value
		cN0 and/or pN0 (n = 100)	pN(+) (n = 34)	
Sex	Male	53	22	.318
	Female	47	12	
Age	≦ 68	51	17	.919
	≧ 69	49	17	
Teeth extraction by previous physicians	None	70	17	.040^a^
	Done	30	17	
T stage	T1・2	55	16	.845
	T3・4	45	18	
Radiographical type	No or Pressure	66	9	<.001^a^
	Moth-eaten	34	25	
Histological pattern	No or Erosive	59	14	.077
	Infiltrative	41	20	
Histological differentiation	Well	64	17	.161
	Moderately/Poorly	36	17	

a Significant at *p* < .05.

**Table 6:** Results of multivariate analysis in risk factors for cervical lymph node metastasis.

Variables	OR (95 % CI)	*p* value
Teeth extraction by previous physicians None/Done	2.078 (1.015–4.973)	.043^a^
Radiographical type No or Pressure type/Moth-eaten type	4.298 (1.733–10.656)	.020^a^

Abbreviations: OR, odds ratio; CI, confidence interval.

a Significant at *p* < .05.

## 4 Discussion

The characteristic feature of mandibular gingival SCC is the ease of mandibular invasion, which is distinct from other primary oral soft tissue tumors, due to its peculiar anatomy [9]. Therefore, surgery is the first choice for treatment of mandibular gingival SCC. The extent of resection of the mandible and method of reconstruction should be considered before planning the surgery [1]. Several prognostic factors for mandibular gingival SCC have been reported in previous studies; as follows, extractions of teeth in the tumor site, T stage, histological differentiation of the primary tumor, histological bone invasion, lymph node metastasis, ENE [[2], [3], [4], [5],^11^]. However, limited number of cases from a single center treated using a consistent approach over time have been reported [11]. We treated 134 patients over a 10-year period, and we believe that the statistical analysis of such considerable number of clinical cases is worthwhile.

In our study, histological cervical lymph node metastasis was a significant poor prognostic factor in mandibular gingival SCC. We further investigated in detail and identified two factors involved in histological cervical lymph node metastasis; teeth extractions by previous physicians and the moth-eaten type of radiographic bone resorption.

Previous studies have reported extractions of teeth by previous physicians before initial treatment in approximately 30 % of mandibular gingival SCC patients [2]. In this study, there was 35.1 % (47/134 cases), which is slightly higher than that reported in previous studies. These teeth are extracted because increased teeth mobility and misdiagnosed as periodontal disease [12]. Although mandibular gingival SCC patients that have been teeth extracted were reported to be poor in prognosis [2], in this study, the 5-year disease-specific survival rate for patients of teeth extraction was 90.7 %, compared to 95.6 % for patients not extracted, which was no significant difference. Even in cases of extraction, there are some reports of a good prognosis with few local recurrences with appropriate decisions on the extent of resection of the mandible [13]. It was suggested that our method of mandibular resection was appropriate for the cases had been teeth extracted by previous physicians. However it is most important to enlighten practicing dentist the differentiation of gingival carcinoma and periodontal disease, and not to extract the teeth if tumor is suspected.

Even in recent reports, poor prognostic factors for mandibular gingival SCC have been reported as primary tumor invasion in the bone marrow [14,15]. In particular, histological infiltrative patterns of bone invasion have been reported to exhibit a significantly poor prognosis [4]. However, if the infiltrative pattern of bone invasion is accurately diagnosed preoperatively, a good outcome can be achieved with the appropriate mandibular resection [16]. In this study, the moth-eaten radiographic type of the mandibular resorption was significantly associated with a histologically infiltrative pattern. In this study, the radiographical type of mandibular resorption was determined on panoramic radiograph. Panoramic radiograph has the advantage of high versatility because it is convenient and available at any institution, however, it has its limitations. These include the inability to measure anatomically accurate distances, the presence of artifacts caused by overlapping three-dimensional structures, and the limited sensitivity of resolution when visualizing small biological changes. Future research should investigate using advanced imaging techniques.

It has been reported that the incidence of cervical lymph node metastasis is high in patients in whom the teeth are extracted before initial treatment or patients with moth-eaten radiographic type of the mandibular resorption, and the prognoses of such patients are poor [2,3]. The results of this study also showed that teeth extractions by previous physicians and the moth-eaten type of radiographic bone resorption were risk factors for cervical lymph node metastasis. In this study, neck dissection was performed in 109 of 134 patients. The reason for this was that the study included a large number of cases in which surgery was performed on the cervical region for primary tumor resection using from cervical approach and immediate reconstruction for the mandible. In other words, 81 of 106 cN0 patients were treated by elective neck dissection. It was suggested that such an aggressive methods of excision might be required for cases with these risk factors. These results are consistent with previous reports [17,18].

This study had some limitations. This retrospective study had an inadequate sample size for multivariate analysis to evaluate risk factors for lymph node metastasis. It was important to reduce the number of cases excluded from this study by proper management of follow-up data. In addition, improving the local control rate would increase the sample size. We believe that additional follow-up and data collection from a larger population would provide further insights into risk factors for cervical lymph node metastasis. In addition, due to the retrospective nature of this study, there was selection bias and lack of control for certain confounding factors. Based on the results of this study, we make the following suggestions for future research. Collecting larger and more diverse samples in collaboration with multicenter institutions, and using more advanced diagnostic imaging techniques for prospective study. This will lead to the selection of even more prognostic risk factors. These suggestions will guide future research in mandibular gingival SCC.

In conclusion, the risk factor for poor prognosis in mandibular gingival SCC was cervical lymph node metastasis. In addition, teeth extractions by previous physicians and moth-eaten radiographic type of mandibular resorption were the risk factors for cervical lymph node metastasis. It is recommended that these cases be treated considering the possibility of cervical lymph node metastasis.

## Data availability statement

Data will be made available on request.

## Funding

This research did not receive any specific grant from funding agencies in the public, commercial, or not-for-profit sectors.

## CRediT authorship contribution statement

**Hirofumi Tomioka:** Conceptualization, Data curation, Formal analysis, Investigation, Methodology, Project administration, Supervision, Validation, Writing – original draft, Writing – review & editing. **Naoto Nishii:** Data curation. **Yu Oikawa:** Data curation. **Takuma Kugimoto:** Data curation. **Takeshi Kuroshima:** Data curation. **Hideaki Hirai:** Data curation. **Kou Kayamori:** Formal analysis. **Atsushi Kaida:** Formal analysis. **Masahiko Miura:** Formal analysis. **Hiroyuki Harada:** Conceptualization, Supervision, Writing – review & editing.

## Declaration of competing interest

The authors declare that they have no known competing financial interests or personal relationships that could have appeared to influence the work reported in this paper.

## References

[1] Takushima A., Harii K., Asato H. (2005). Choice of osseous and osteocutaneous flaps for mandibular reconstruction. Int. J. Clin. Oncol..

[2] Suzuki K., Shingaki S., Nomura T. (1998). Oral carcinoma detected after extraction of teeth: a clinical and radiographic analysis of 32 cases with special reference to metastasis and survival. Int. J. Oral Maxillofac. Surg..

[3] Nakayama E., Yoshiura K., Yuasa K. (2000). A study of the association between the prognosis of carcinoma of the mandibular gingiva and the pattern of bone destruction on computed tomography. Dentomaxillofacial Radiol..

[4] Shaw R.J., Brown J.S., Woolgar J.A. (2004). The influence of the pattern of mandibular invasion on recurrence and survival in oral squamous cell carcinoma. Head Neck.

[5] Safi A.F., Kauke M., Grandoch A. (2018). The importance of lymph node ratio for patients with mandibular infiltration of oral squamous cell carcinoma. J. Cranio-Maxillo-Fac. Surg..

[6] Ebrahimi A., Murali R.K., Gao K. (2011). The prognostic and staging implications of bone invasion in oral squamous cell carcinoma. Cancer.

[7] Izumo T., Kirita T., Ariji E. (2012). General rules for clinical and pathological studies on oral cancer: a synopsis. Jpn. J. Clin. Oncol..

[8] Okura M., Yanamoto S., Umeda M. (2016). Prognostic and staging implications of mandibular canal invasion in lower gingival squamous cell carcinoma. Cancer Med..

[9] Swearingen A.G., McGraw J.P., Palumbo V.D. (1966). Roentgenographic pathologic correlation of carcinoma of the gingiva involving the mandible. Am. J. Roentgenol. Radium Ther. Nucl. Med..

[10] Slootweg P.J., Muller H. (1989). Mandibular invasion by oral squamous cell carcinoma. J. Cranio-Maxillo-Fac. Surg..

[11] Overholt S.M., Eicher S.A., Wolf P. (1996). Prognostic factors affecting outcome in lower gingival carcinoma. Laryngoscope.

[12] I T Miura K., Kakehashi H. (2021). Carcinoma cuniculatum of the lower gingiva masked with leukoplakia: a case of difficult diagnosis. J Oral Maxillofac Surg Med Pathol.

[13] Choi E.J., Zhang X., Kim H.J. (2011). Prognosis of gingival squamous cell carcinoma diagnosed after invasive procedures. Asian Pac. J. Cancer Prev. APJCP.

[14] Du W., Fang Q., Wu Y. (2019). Oncologic outcome of marginal mandibulectomy in squamous cell carcinoma of the lower gingiva. BMC Cancer.

[15] Niu L.X., Feng Z.E., Wang D.C. (2017). Prognostic factors in mandibular gingival squamous cell carcinoma: a 10-year retrospective study. Int. J. Oral Maxillofac. Surg..

[16] Totsuka Y., Usui Y., Tei K. (1991). Mandibular involvement by squamous cell carcinoma of the lower alveolus: analysis and comparative study of histologic and radiologic features. Head Neck.

[17] Gou L., Yang W., Qiao X. (2018). Marginal or segmental mandibulectomy: treatment modality selection for oral cancer: a systematic review and meta-analysis. Int. J. Oral Maxillofac. Surg..

[18] Harada H., Shimamoto H., Oikawa Y. (2021). Mandibular reconstruction with scapular systems: a single-center case series involving 208 flaps. Plast. Reconstr. Surg..

